# Lentinan alleviates metabolic dysfunction implicating *Parabacteroides goldsteinii*-enriched gut microbiota and hepatic lipid metabolism reprogramming through gut-liver axis-associated mechanisms

**DOI:** 10.3389/fnut.2026.1841358

**Published:** 2026-07-01

**Authors:** Demin Cao, Liwei Huang, Xinyue Zhang, Xinyu Zhang, Zhiwen Zhao, Xidai Long, Xiaoying Zhu, Yulei Li

**Affiliations:** 1 Clinicopathological Diagnosis and Research Center, The Affiliated Hospital of Youjiang Medical University for Nationalities, Baise, China; 2 Key Laboratory of Molecular Pathology for Hepatobiliary Diseases of Guangxi, Baise, China; 3 Clinical Medical School, Youjiang Medical University for Nationalities, Baise, Guangxi, China

**Keywords:** gut microbiota, hepatic lipid synthesis, high-fat diet, lentinan, metabolic dysfunction

## Abstract

Metabolic disorders represent a global health challenge requiring novel therapeutic strategies targeting the gut-liver axis. This study investigates the protective effects and mechanisms of lentinan, a bioactive polysaccharide from *Lentinus edodes*, against high-fat diet (HFD)-induced metabolic dysfunction. HFD-fed mice were treated with lentinan. Comprehensive phenotypic assessments, metagenome sequencing, hepatic transcriptomics, and correlation analyses were performed to elucidate mechanisms. Lentinan intervention significantly ameliorated dyslipidemia, hepatic steatosis, systemic inflammation, and intestinal barrier dysfunction in HFD-fed mice. Mechanistically, lentinan induced taxonomically selective gut microbiota remodeling, characterized by substantial enrichment of *Parabacteroides goldsteinii* (positively correlated with hepatic *Plppr3* expression) and reduction of *Romboutsia ilealis* (negatively correlated with *Dgkh* and *Nfat5*), while paradoxically decreasing *Akkermansia muciniphila* despite metabolic improvements. Hepatic transcriptomics revealed significant downregulation of glycerolipid metabolism and oxidative phosphorylation pathways, directly correlating with reduced lipid accumulation and improved serum biochemistry. Unlike conventional prebiotics, lentinan functions as a precision modulator of specific microbial metabolic functions, particularly L-arginine and uridine 5′-monophosphate (UMP) biosynthesis pathways, which interface with host inflammatory and lipid metabolism. These findings establish lentinan as a promising therapeutic candidate for metabolic syndrome management through coordinated gut microbiota-liver axis modulation, providing a conceptual framework for developing precision microbiome-targeted interventions.

## 1 Background

Obesity has emerged as one of the most pressing global public health challenges of the 21st century. According to the World Health Organization (WHO) statistics, the global adult obesity rate has surpassed 16% in 2022, with an exponential rise in childhood and adolescent obesity (1). Obesity is not only a primary driver of cardiovascular diseases, type 2 diabetes, and non-alcoholic fatty liver disease (NAFLD) but also strongly linked to chronic low-grade inflammation, insulin resistance, and multi-organ metabolic dysfunction (2, 3). HFD serve as a critical catalyst for obesity development, inducing energy imbalance, adipose tissue expansion, and systemic inflammatory responses. For instance, HFD activate endoplasmic reticulum stress and oxidative stress pathways, leading to the release of pro-inflammatory cytokines (e.g., IL-6, TNF-α) and exacerbating metabolic disturbances (4). However, current interventions for HFD-induced metabolic diseases remain limited to pharmacological treatments and lifestyle modifications (5), necessitating the exploration of safer, sustainable therapeutic strategies.

The gut microbiota plays a pivotal role in regulating host metabolism and immune homeostasis. Obesity is consistently linked to reduced microbial *α*-diversity and shifts in specific taxa or functional pathways—rather than a universal increase in the Firmicutes-to-Bacteroidetes ratio, which shows poor reproducibility in human studies. HFD can enrich certain *Bacteroides* species (e.g., *Bacteroides uniformis*, *Bacteroides thetaiotaomicron*) in some dietary contexts (6, 7), but their metabolic impact stems not from excessive short-chain fatty acid (SCFA) production—primarily a function of *Clostridia*—but from bile acid modification, lipopolysaccharide (LPS) release, and mucin degradation (8, 9). Microbial metabolites such as SCFAs, tryptophan derivatives, and secondary bile acids modulate host physiology via receptors like GPR43/41, AhR, FXR, and TGR5 (10, 11). Dysbiosis disrupts these pathways, compromises gut barrier integrity, promotes LPS translocation, and drives chronic low-grade inflammation, thereby contributing to insulin resistance and NAFLD. These mechanisms underpin the gut–liver axis and support microbiota-targeted therapeutic strategies.

Lentinan, a β-glucan polysaccharide extracted from the edible mushroom *Lentinula edodes*, is renowned for its immunomodulatory and anti-inflammatory properties. Preclinical studies show that lentinan enhances innate and adaptive immunity by activating macrophages, T cells, and dendritic cells (12). It also mitigates chronic inflammation by inhibiting the nuclear factor-κB (NF-κB) pathway and reducing pro-inflammatory cytokine production (e.g., IL-6, TNF-α) (13). In metabolic diseases, lentinan has been shown to ameliorate HFD-induced insulin resistance and fatty liver (14). However, its mechanisms remain incompletely understood. Current research primarily focuses on its immune-regulatory effects, with a critical gap in systematic investigations of how lentinan modulates the gut-liver axis to improve obesity-related metabolic disorders. Unraveling this interplay could not only reveal its therapeutic value but also inform the development of microbiota-targeted natural interventions. This study integrates metagenomic, transcriptomic, and phenotypic data to systematically dissect lentinan’s modulation of the obesity-related gut-metabolism axis. Our findings aim to provide mechanistic insights and inform microbiota-targeted therapies for metabolic diseases.

## 2 Materials and methods

### 2.1 Experiment design

Four-week-old male C57BL/6J mice were obtained from Beijing Vital River Laboratory Animal Technology Co., Ltd. (China). Animals were housed in groups of four to five per cage with free access to food and sterile reverse osmosis drinking water under controlled environmental conditions (37 °C ± 2 °C, 12-h dark–light cycle). Following a one-week acclimatization period, mice were randomly assigned to three experimental groups (*n* = 7 per group) and fed for 13 weeks with either standard chow diet (LFD with 1 group, 13.5% of energy from fat; Cat. No. 1022, Huafukang, China) or high-fat diet (HFD with 2 groups; 60% of energy from fat; Cat. No. H10060, Huafukang, China). Mice received daily intragastric administration of 100 μL sterile saline (vehicle control, LFD/HFD) or lentinan (100 mg/kg body weight; Cat. No. S23110, OriLeaf, China, HFD + Lentinan) dissolved in saline. At the experimental endpoint, non-fasted animals were anesthetized and whole blood was collected by cardiac puncture. Epididymal white adipose tissue (eWAT) and liver were immediately harvested, weighed, snap-frozen in liquid nitrogen, and stored at −80 °C for subsequent analyses.

### 2.2 Oral glucose tolerance test (OGTT)

The OGTT was performed at week 12 of the intervention. After overnight fasting, mice were administered an oral glucose (2 g/kg body weight) by oral gavage. Blood glucose levels were measured using a glucose meter (Accu-Chek Performa, Roche, Switzerland) at baseline (0 min) and at 30, 60, and 120 min post-glucose administration. The area under the curve (AUC) for glucose tolerance was calculated using GraphPad Prism software (v10.5.0).

### 2.3 Histological analysis

Liver and colon samples were fixed in 10% formaldehyde phosphate-buffered saline (pH 7.4), embedded in paraffin, sectioned at 5 μm thickness, and stained with hematoxylin and eosin (H&E) and Alcian Blue. All histological assessments were performed using a DM300 light microscope (Leica, Germany). Tissue sections were randomized and evaluated by a board-certified veterinary pathologist who was blinded to the experimental groups. For immunohistochemical analysis of Claudin-1, five colon tissue sections per group were randomly selected and processed using a polyclonal anti-Claudin-1 antibody (Proteintech, 28674-1-AP). Positive staining areas were quantified using ImageJ software (version 1.54 g) by calculating the percentage of positively stained regions relative to the total tissue area.

### 2.4 Biochemical and cytokine measurements

Whole blood was collected by cardiac puncture into tubes without anticoagulant, allowed to clot for 30 min at room temperature, and centrifuged at 15,000 × *g* for 1 min. The resulting serum was used to measure total cholesterol (TC), high-density lipoprotein cholesterol (HDL-C), low-density lipoprotein cholesterol (LDL-C), lipase, alanine aminotransferase (ALT), aspartate aminotransferase (AST), cholinesterase (CHE), and lactate dehydrogenase (LDH) using commercial assay kits according to the manufacturers’ protocols (Elabscience E-BC-K235-M, E-BC-K236-M, E-BC-K052-M, E-BC-K046-M). Serum interleukin-6 (IL-6) and tumor necrosis factor-alpha (TNF-α) concentrations were quantified using enzyme-linked immunosorbent assay (ELISA) kits (Cat. No. EK2153EG and EK282EG, respectively; Multi Science, China).

### 2.5 Metagenome sequencing and analysis

At week 13, mice were euthanized under anesthesia. Colonic luminal contents were immediately collected, snap-frozen in liquid nitrogen, and stored at −80 °C until DNA extraction for metagenomic sequencing. Genomic DNA was extracted from fecal samples using the cetyltrimethylammonium bromide (CTAB)/sodium dodecyl sulfate (SDS) method. Sequencing libraries were prepared following the manufacturer’s protocol and subjected to paired-end sequencing on an Illumina HiSeq2500 platform (Novogene Technology Co. Ltd., Tianjin, China). Raw reads were quality-filtered using Trimmomatic (v0.33), and host DNA sequences were removed by alignment to the mouse reference genome (GRCm39) using bowtie2 (v2.5.1). Taxonomic classification was performed with MetaPhlAn4 (v4.1.1) (15). Alpha and beta diversity analyses were conducted using the vegan package (v2.6) in R (v4.5.1), with statistical significance assessed by analysis of similarities (ANOSIM) based on Bray-Curtis and Jaccard dissimilarity matrices. Microbial functional profiling was performed using HUMAnN3 (v3.9) (16), and differentially abundant taxa and pathways were identified using LEfSe (v 1.1.01) (17). Raw sequencing data were deposited in the China National Center for Bioinformation (CNCB) Genome Sequence Archive (GSA) database (18) (Accession No. CRA036704) that are publicly accessible at https://ngdc.cncb.ac.cn/gsa.

### 2.6 RNA sequencing and transcriptome analysis

Liver tissues were harvested, flash-frozen, and used for RNA extraction at week 13. Total RNA was extracted using the phenol-chloroform method. Polyadenylated RNA was purified using poly-T oligo-attached magnetic beads, and stranded RNA-seq libraries were constructed from 2 μg of RNA per sample. High-throughput sequencing was performed using DNBSEQ-T7 PE150 technology (Novogene Technology Co. Ltd., Tianjin, China). Clean reads were aligned to the mouse reference genome (GRCm39) using HISAT2 (v2.2.1) (19), and gene expression levels were quantified as transcripts per kilobase million (TPM) using StringTie (v2.2.1) (20) with GENCODE vM33 annotation. Differential gene expression analysis was conducted using DESeq2 (v1.46.0), with differentially expressed genes (DEGs) defined as those with |log_2_ fold change| ≥ 1 and false discovery rate (FDR) ≤ 0.05. Gene Ontology (GO) and Kyoto Encyclopedia of Genes and Genomes (KEGG) enrichment analyses were performed using the clusterProfiler package (v4.17.0) (21) in R (v4.5.1). RNA-seq data were deposited in the CNCB GSA database (Accession No. CRA036737).

### 2.7 Quantitative real-time polymerase chain reaction (qRT-PCR)

Total RNA was extracted from liver tissues following the same protocol as described in the transcriptome section. First-strand cDNA synthesis was performed using the ToloScript All-in-one RT EasyMix (Tolobio, China) according to the manufacturer’s instructions. Quantitative real-time PCR was conducted with SYBR qPCR Master Mix (Tolobio, China) on a real-time PCR system, with glyceraldehyde-3-phosphate dehydrogenase (GAPDH) serving as the internal reference gene for normalization. The primer sequences used for amplification are listed in Supplementary Table S4.

### 2.8 Microbiome-transcriptome integrative analysis

Comprehensive correlation analyses were performed between differentially abundant gut microbial taxa, microbial metabolic pathways, and hepatic differentially expressed genes using Spearman’s rank correlation method implemented in the psych package (v2.4.6) in R. Correlation results were visualized as heatmaps using the pheatmap package (v1.0.12) and as networks using the igraph package (v2.1.2).

### 2.9 Statistical analysis

All statistical analyses were performed using GraphPad Prism software (v10.5.0; San Diego, CA, USA). Data are presented as mean ± standard error of the mean (SEM). Differences between two groups were assessed using unpaired two-tailed Student’s *t*-test or Mann–Whitney U test for non-normally distributed data. Comparisons among three or more groups were performed using one-way ANOVA followed by Dunnett’s *post hoc* test. A *p*-value ≤ 0.05 was considered statistically significant. Statistical significance was defined as *p ≤ 0.05, **p ≤ 0.01, ***p ≤ 0.001, and ****p ≤ 0.0001, with “ns” indicating no significant difference.

## 3 Results

### 3.1 Lentinan alleviated high-fat diet-induced metabolic dysfunction

**Figure 1:**
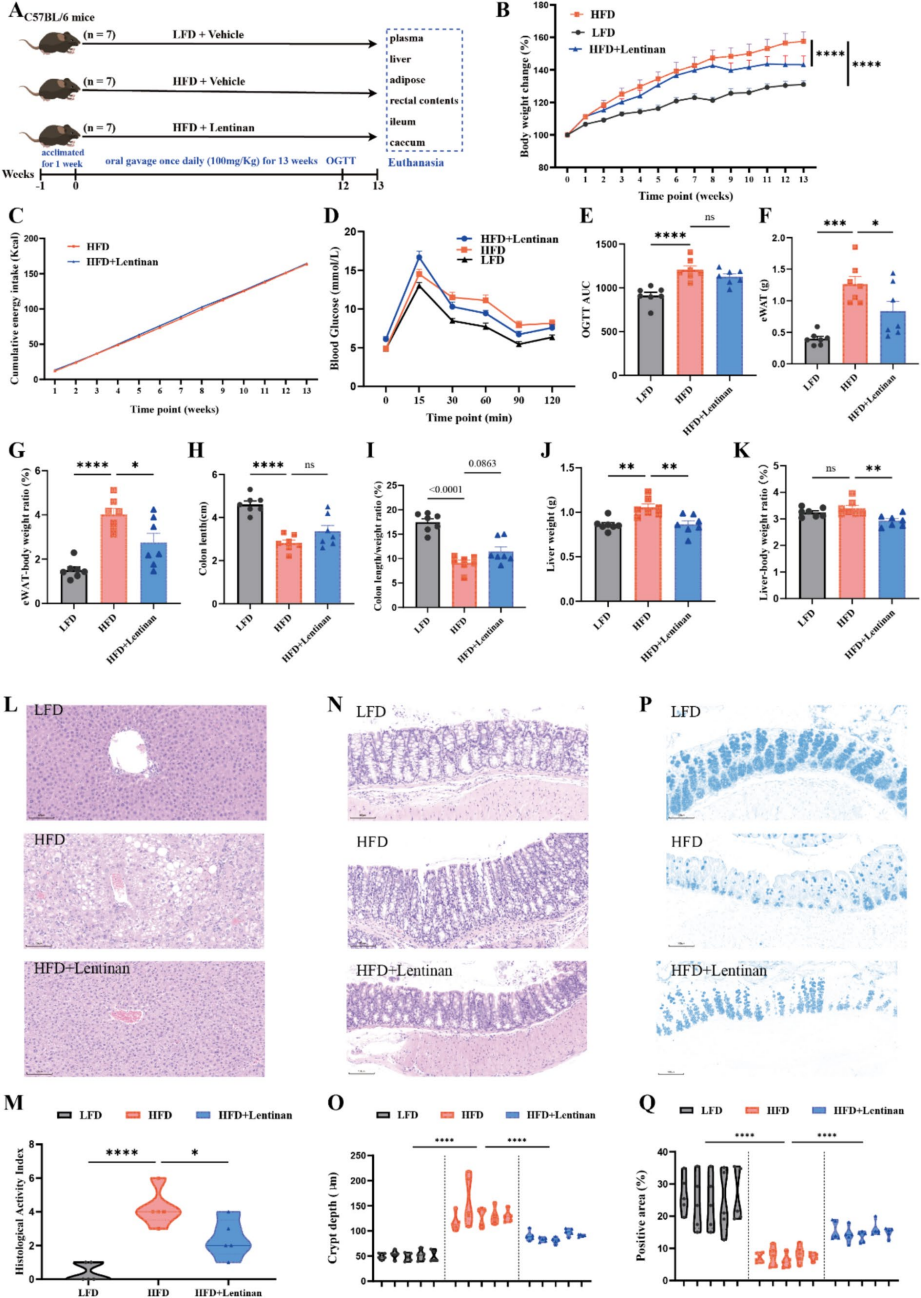
Lentinan alleviated high-fat diet-induced metabolic dysfunction in mice. **(A)** Schematic representation of the experimental design. Mice were divided into three groups: low-fat diet (LFD), high-fat diet (HFD), and HFD supplemented with lentinan (HFD + Lentinan). **(B)** Body weight changes. **(C)** Cumulative of energy intake of each mouse. Oral glucose tolerance test (OGTT) curves **(D)** and AUC of OGTT **(E)**. Comparison of epididymal white adipose tissue (eWAT) weight **(F)** and eWAT-body weight ratio **(G)**. Comparison of colon length **(H)** and colon length/body weight ratio **(I)**. Comparison of liver weight **(J)** and liver-body weight ratio **(K)**. **(L)** Liver H&E staining images. **(M)** Quantification of liver pathology scores based on H&E staining. **(N)** Colon H&E staining images. **(O)** Crypt depth analysis from colon H&E staining. **(P)** Alcian Blue staining of colon sections. **(Q)** Quantification of Alcian Blue positive areas. Data are presented as the mean ± SEM. *n* = 7 mice per group. Statistical analysis was performed using one-way ANOVA followed by the Dunnett’s*post hoc* test. *p ≤ 0.05; **p ≤ 0.01; ***p ≤ 0.001; ****p ≤ 0.0001.

To evaluate the impact of lentinan on HFD-induced metabolic dysfunction, we conducted a comprehensive study involving three groups: LFD, HFD, and HFD supplemented with lentinan (HFD + Lentinan) (Figure 1A). Our results indicate that chronic lentinan administration significantly attenuated multiple pathological manifestations of HFD-induced metabolic dysfunction in mice.

After a 13-week intervention period, HFD-fed mice exhibited markedly elevated body weight gain exceeding 20% relative to the LFD group, whereas lentinan co-administration substantially suppressed this weight gain (*p* ≤ 0.0001; Figure 1B). Cumulative energy intake remained comparable between HFD and HFD + lentinan groups (Figure 1C), indicating that the anti-obesity effect was independent of altered food consumption. Although lentinan induced a transient reduction in blood glucose at 30 min during the oral glucose tolerance test, no significant difference was observed in the overall glucose excursion curve or area under the curve (AUC) compared to the HFD group (Figures 1D,E).

Lentinan intervention significantly reduced epididymal white adipose tissue mass (*p* ≤ 0.05; Figure 1F) and lowered the epididymal fat pad-to-body weight ratio (Figure 1G). Hepatic parameters were notably improved: absolute liver weight and liver-to-body weight ratio were both significantly decreased in the lentinan-treated group (*p* ≤ 0.01 for both; Figures 1J,K), reaching levels comparable to the LFD control. Histopathological analysis of liver sections revealed extensive lipid vacuolization in HFD-fed mice, which was markedly ameliorated by lentinan treatment (Figure 1L). Correspondingly, the semi-quantitative liver pathology score was significantly reduced (*p* ≤ 0.05; Figure 1M).

In the colon, HFD feeding induced significant shortening of colon length relative to the LFD group, with lentinan showing a modest ameliorative trend (Figures 1H,I). H&E staining demonstrated that HFD severely disrupted colonic epithelial architecture, while lentinan preserved mucosal integrity (Figure 1N). Crypt depth, which was significantly increased in the HFD group (*p* ≤ 0.001), was restored to near-normal levels by lentinan intervention (*p* ≤ 0.001; Figure 1O). Alcian Blue staining further revealed that HFD markedly depleted goblet cells and reduced mucin-positive areas (p ≤ 0.001), whereas lentinan effectively preserved goblet cell morphology and significantly restored the mucin-positive area ratio (*p* ≤ 0.001; Figures 1P,Q).

Collectively, these findings demonstrate that lentinan exerts beneficial effects on multiple facets of metabolic health, including body weight management, adiposity reduction, improved liver function, and enhanced intestinal integrity. These results suggest that lentinan may serve as a promising therapeutic agent for combating HFD-induced metabolic disorders.

### 3.2 Lentinan reduces inflammatory markers and improves hepatic function

To further elucidate the molecular mechanisms underlying the effects of HFD on mice and the beneficial role of lentinan, we assessed various metabolic and chronic inflammation-related biomarkers at the molecular level.

**Figure 2:**
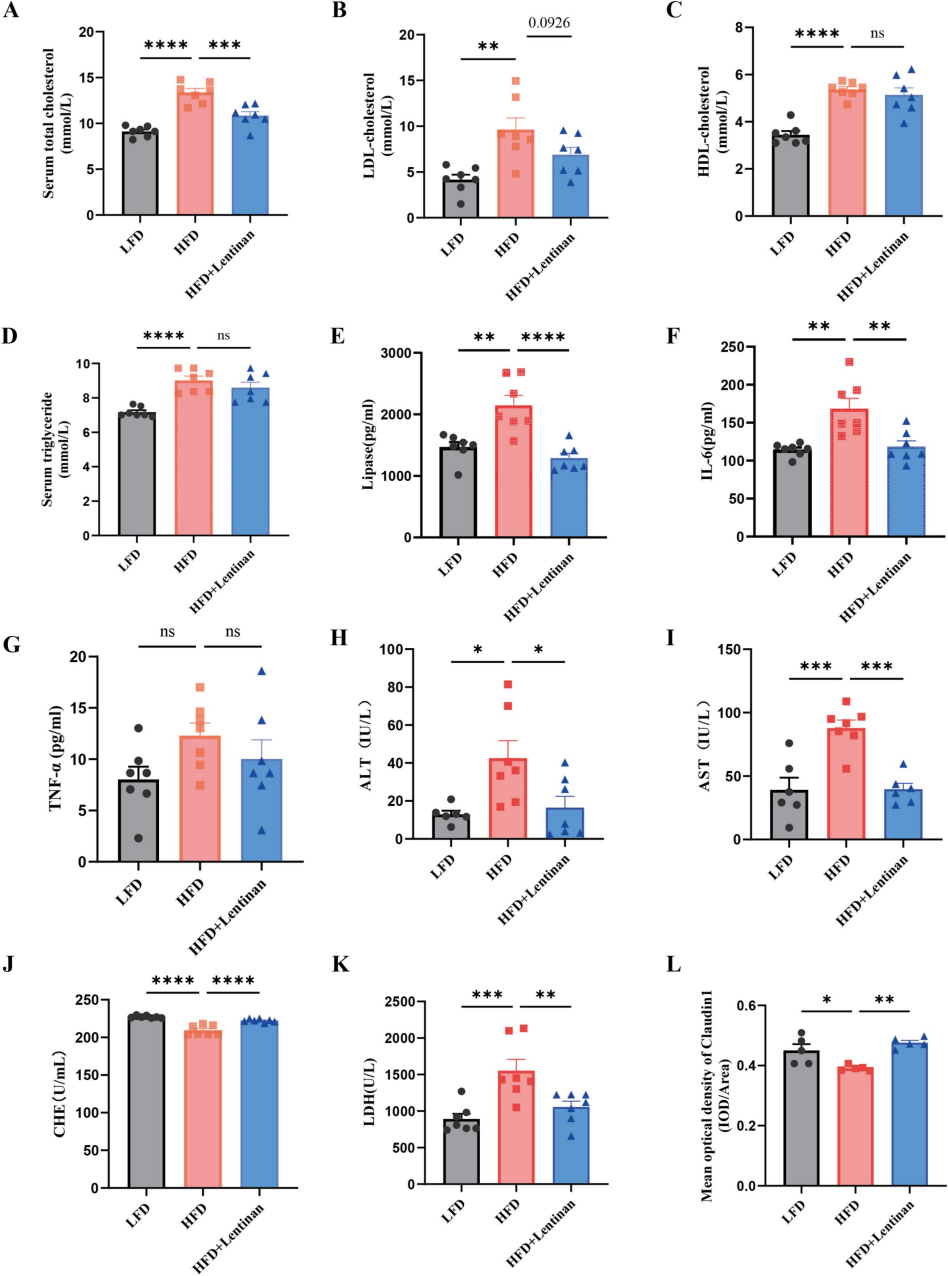
Lentinan reduces inflammatory markers and improves liver function. **(A)** Serum total cholesterol levels. **(B)** Serum low-density lipoprotein (LDL) levels. **(C)** Serum high-density lipoprotein (HDL) levels. **(D)** Serum triglycerides. **(E)** Serum lipase levels. **(F)** Serum IL-6 levels. **(G)** Serum TNF-α levels. **(H)** Serum alanine aminotransferase (ALT) levels. **(I)** Serum aspartate aminotransferase (AST) levels. **(J)** Serum cholinesterase (CHE) levels. **(K)** Serum lactate dehydrogenase (LDH) levels. **(L)** Immunohistochemical analysis of claudin-1 in colon tissue (*n* = 5). Statistical analysis was performed using one-way ANOVA followed by the Dunnett’s *post hoc* test. *p ≤ 0.05; **p ≤ 0.01; ***p ≤ 0.001; ****p ≤ 0.0001.

In order to evaluate the impact of lentinan on lipid metabolism, we measured serum total cholesterol levels. As shown in Figure 2A, HFD-fed mice exhibited a significant increase in total cholesterol compared to the LFD group (*p* ≤ 0.0001). Lentinan treatment effectively reduced this elevation (*p* ≤ 0.001). Similarly, serum low-density lipoprotein (LDL) levels were significantly elevated in the HFD group, and lentinan showed a trend towards normalizing these levels, although the difference did not reach statistical significance (*p* = 0.0926) (Figure 2B). In contrast, no significant changes were observed in high-density lipoprotein (HDL) levels following lentinan treatment (Figure 2C). Serum triglyceride accumulation was not significantly ameliorated by Lentinan treatment (Figure 2D), likely due to the overwhelming influence of continued high-fat dietary intake on TG homeostasis. Additionally, serum lipase levels were significantly lower in the lentinan-treated group (*p* ≤ 0.0001) (Figure 2E), indicating an improvement in lipid metabolism.

We also examined inflammatory markers to assess the anti-inflammatory effects of lentinan. Serum interleukin-6 (IL-6) levels were significantly reduced in the lentinan-treated group compared to the HFD group (*p* ≤ 0.01) (Figure 2F). Although there was a trend towards reduced tumor necrosis factor-alpha (TNF-α) levels in the lentinan-treated group, the difference did not reach statistical significance (Figure 2G). These findings suggest that lentinan has potent anti-inflammatory properties. Furthermore, to investigate the effect of lentinan on liver function, we measured serum alanine aminotransferase (ALT) and aspartate aminotransferase (AST) levels. Both ALT and AST levels were significantly lower in the lentinan-treated group compared to the HFD group (*p* ≤ 0.05 for ALT and *p* ≤ 0.001 for AST) (Figures 2H,I), indicating improved liver health. Further analysis revealed that lentinan significantly increased serum cholinesterase (CHE) levels (*p* ≤ 0.0001) (Figure 2J), bringing them to levels comparable to the LFD group, suggesting enhanced liver synthetic function. Moreover, serum lactate dehydrogenase (LDH) levels, which are often elevated in response to tissue damage or inflammation, were significantly reduced by lentinan treatment (*p* ≤ 0.01) (Figure 2K). This reduction in LDH levels further supports the hepatoprotective effects of lentinan. Finally, we evaluated the integrity of the intestinal barrier by assessing claudin-1 expression in colon tissue. Immunohistochemical analysis revealed that lentinan treatment significantly increased claudin-1 expression (*p* ≤ 0.01) (Figure 2L), suggesting enhanced intestinal barrier function.

Collectively, these results demonstrate that lentinan not only mitigates lipid metabolic disturbances and reduces systemic inflammation but also improves liver function and enhances intestinal barrier integrity, underscoring its potential as a therapeutic agent against HFD-induced metabolic disorders.

### 3.3 Lentinan altered gut microbiota composition and enhances metabolic pathways

To investigate the impact of lentinan on the gut microbiota structure and function in HFD-fed mice, we performed metagenomic sequencing of fecal samples.

**Figure 3:**
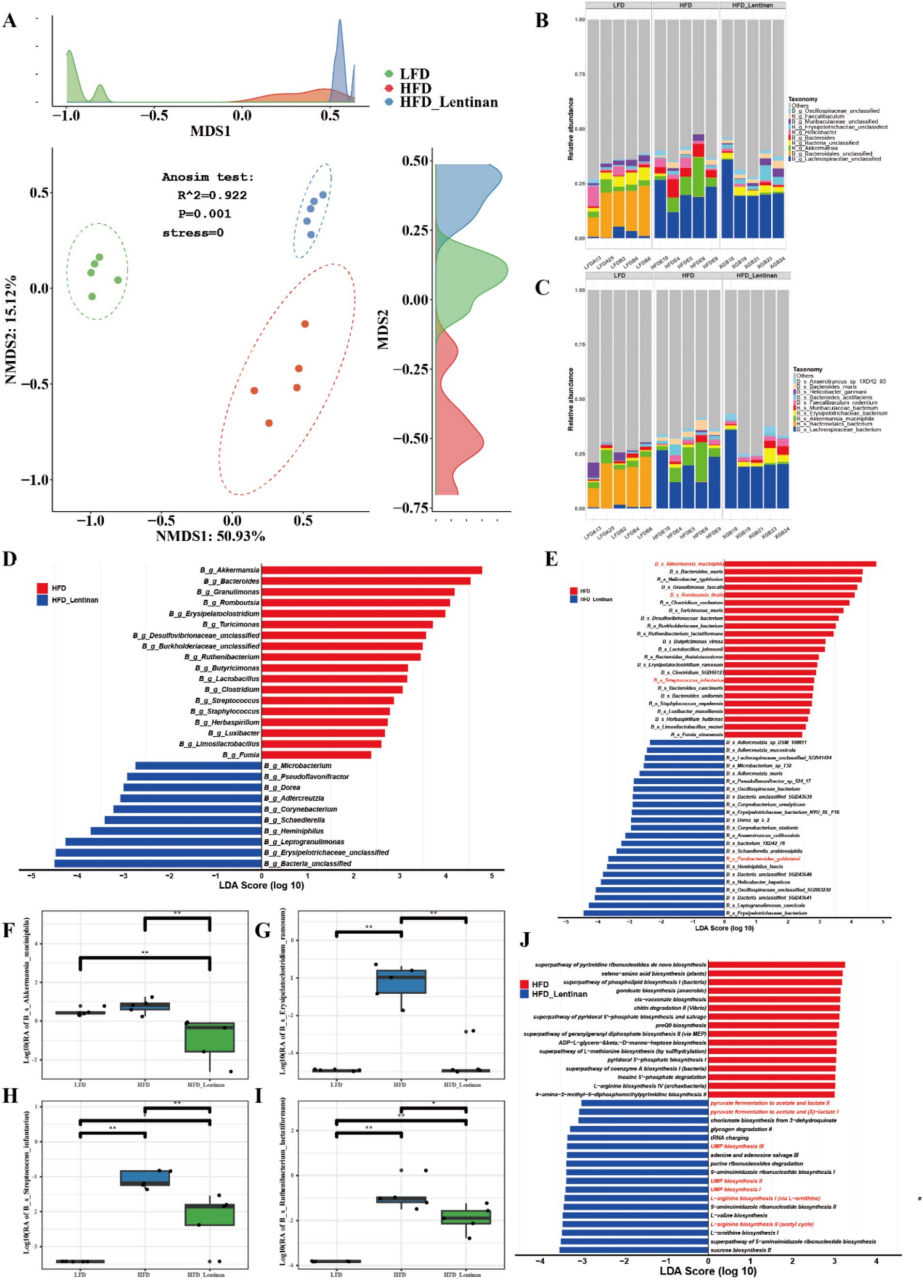
Lentinan altered the structure and function of gut microbiota in HFD mice. **(A)** NMDS analysis based on Jaccard distance for microbial community composition. **(B)** Relative abundance of top 10 genera. **(C)** Relative abundance of top 10 species. **(D)** LEfSe analysis at the genus level. **(E)** LEfSe analysis at the species level. **(F)** Comparison of *Akkermansia muciniphila* abundance among groups. **(G)** Comparison of *Erysipelatoclostridium ramosum* abundance. **(H)** Comparison of *Streptococcus infantarius* abundance. **(I)** Comparison of *Ruthenibacterium lactatiformans* abundance. **(J)** LEfSe analysis of gut microbiota gene metabolic pathways. Statistical analysis was performed using one-way ANOVA followed by the Dunnett’s *post hoc* test. *p ≤ 0.05; **p ≤ 0.01.

In order to assess changes in microbial community composition, we conducted NMDS analysis based on Jaccard distance (Figure 3A). This revealed distinct clustering among the LFD, HFD, and lentinan-treated groups (*p* = 0.001), indicating that lentinan significantly altered the gut microbiota structure. However, the microbial structure did not revert to that of the LFD group, suggesting a unique configuration induced by lentinan. Notably, alpha diversity analysis revealed no significant differences in Shannon, Simpson, or Pielou indices across groups, except for reduced observed species richness under HFD conditions (Supplementary Figure S1), indicating that lentinan primarily modulates community composition rather than overall diversity.

At the genus and species levels, we observed significant shifts in microbial composition under HFD conditions (Figures 3B,C; Supplementary Table S1). Specifically, there was a shift from dominance of *Bacteroidales* to *Lachnospiraceae*. Notably, *Akkermansia muciniphila* was decreased upon lentinan treatment, while *Erysipelotrichaceae* increased. Further analysis using LEfSe identified several differentially abundant taxa at both genus (Figure 3D) and species (Figure 3E) levels. Under HFD conditions, taxa such as *Akkermansia* (*Akkermansia muciniphila*), *Bacteroides* (*Bacteroides muris*), *Granulimonas* (*Granulimonas faecalis*), and *Romboutsia* (*Romboutsia ilealis*) were enriched. Conversely, lentinan treatment induced the enrichment of *Erysipelotrichaceae*, *Leptogranulimonas* (*Leptogranulimonas caecicola*), unclassified bacteria (SGB43541, SGB43546), and beneficial *Parabacteroides goldsteinii*.

Specific comparisons of key taxa revealed that lentinan significantly reduced the abundance of *A. muciniphila* compared to both LFD and HFD groups (*p* ≤ 0.01), while no significant difference showed between HFD and LFD groups (Figure 3F). Additionally, the abundance of *Erysipelatoclostridium ramosum* was significantly higher in the HFD group compared to the other two groups (*p* ≤ 0.01) (Figure 3G). Lentinan also significantly reduced the levels of *Streptococcus infantarius* (*p* ≤ 0.01) (Figure 3H) and *Ruthenibacterium lactatiformans* (*p* ≤ 0.01) (Figure 3I).

Finally, we examined the functional pathways of the gut microbiota using LEfSe analysis (Figure 3J; Supplementary Table S2). Lentinan treatment led to significant increases in pathways such as L-arginine biosynthesis I/II, UMP (uridine 5′-monophosphate) biosynthesis I/II/III, and pyruvate fermentation to acetate and lactate I/II. These findings suggest that lentinan not only alters the microbial community structure but also enhances specific metabolic functions, potentially contributing to its beneficial effects on host health.

### 3.4 Lentinan reprograms hepatic gene expression to attenuate lipid accumulation

In order to elucidate the molecular mechanisms by which lentinan improves hepatic lipid metabolism, we performed transcriptome analysis on liver tissues from HFD-fed mice with or without lentinan supplementation.

**Figure 4:**
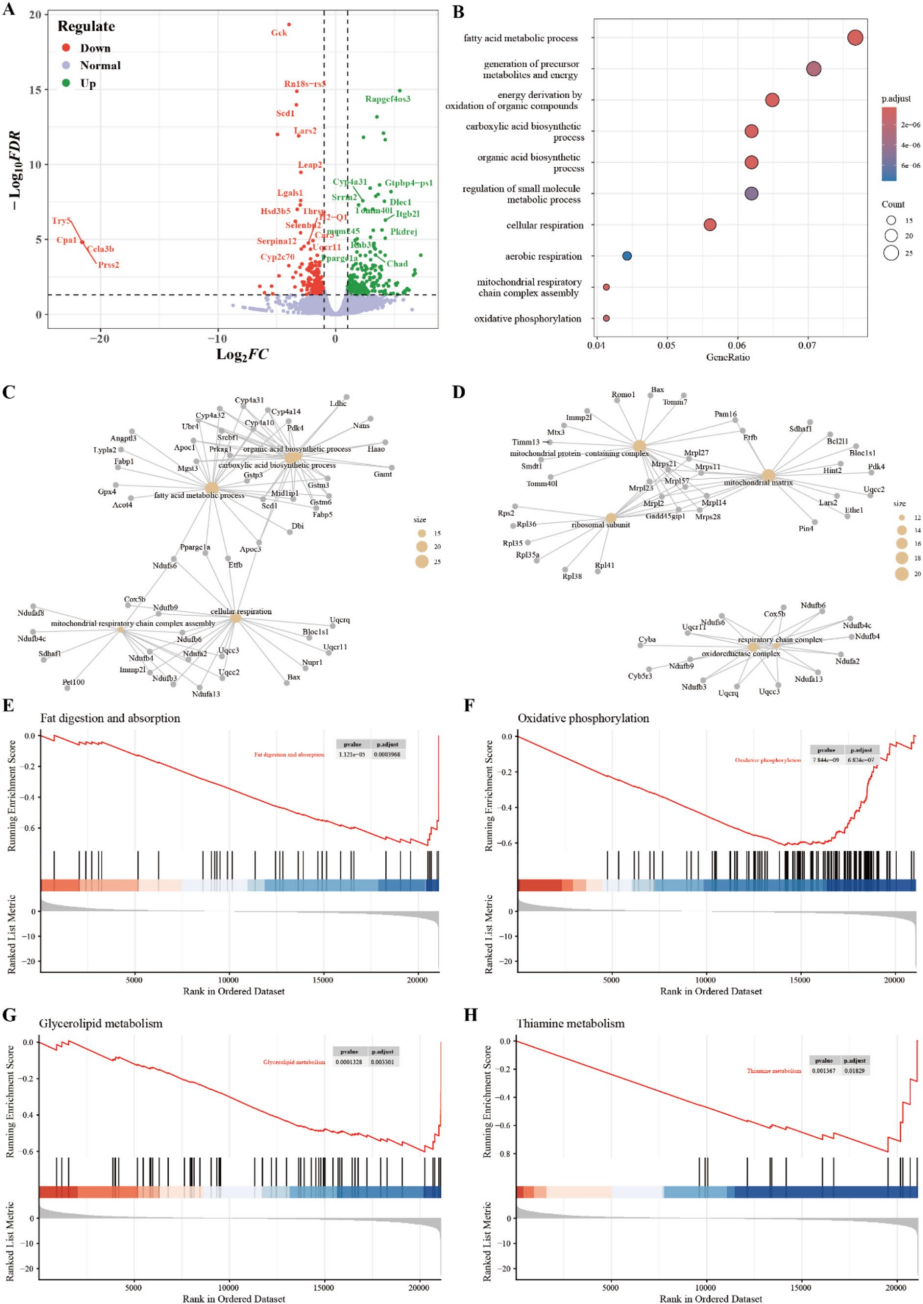
Lentinan modulates hepatic transcriptome to suppress lipid metabolism pathways. **(A)** Volcano plot showing differential gene expression analysis in liver tissues between the lentinan-treated group and HFD group (|log2^FC^| ≥ 1, *p* ≤ 0.05). **(B)** Gene Ontology (GO) enrichment analysis of DEGs. Lentinan treatment significantly altered the expression of genes involved in fatty acid metabolic processes, generation of precursor metabolites and energy, mitochondrial respiratory chain complex assembly, cellular respiration and so forth. **(C)** GO enrichment network analysis for biological processes. **(D)** GO enrichment network analysis for cellular components. **(E–H)** Gene Set Enrichment Analysis (GSEA) based on KEGG pathways. Lentinan treatment induced overall downregulation of genes in the pathways of fat digestion and absorption **(E)**, oxidative phosphorylation **(F)**, glycerolipid metabolism **(G)**, and thiamine metabolism **(H)**.

Comparative analysis of hepatic gene expression profiles revealed 453 differentially expressed genes (DEGs) between the lentinan-treated group and the HFD group, comprising 208 significantly upregulated genes and 245 significantly downregulated genes (Figure 4A; Supplementary Table S3). To validate the RNA-seq findings, we performed qPCR on a subset of differentially expressed genes (*Nfat5, Plppr3, Dgkh, Cpt1a, Lepr*, and *Pparg*) using the original RNA samples. The qPCR results confirmed the expression trends observed in the RNA-seq data (Supplementary Figure S2). Gene Ontology (GO) enrichment analysis demonstrated that these DEGs were significantly associated with fatty acid metabolic processes, generation of precursor metabolites and energy, mitochondrial respiratory chain complex assembly, and cellular respiration (Figure 4B), suggesting that lentinan primarily modulates energy metabolism pathways in the liver. This was further supported by GO enrichment network analysis at both biological process (Figure 4C) and cellular component (Figure 4D) levels.

Gene Set Enrichment Analysis (GSEA) based on KEGG pathways revealed that lentinan treatment induced comprehensive downregulation of multiple lipid metabolism-related pathways. Specifically, genes involved in fat digestion and absorption were significantly suppressed (Figure 4E), indicating reduced lipid uptake and processing in hepatocytes. Similarly, oxidative phosphorylation pathways were downregulated (Figure 4F), suggesting altered energy utilization in the liver. Most notably, glycerolipid metabolism pathways were substantially suppressed by lentinan treatment (Figure 4G), directly linking to reduced triglyceride synthesis and hepatic steatosis. Additionally, thiamine metabolism was also downregulated (Figure 4H), which may contribute to the overall metabolic reprogramming induced by lentinan.

Collectively, these transcriptomic findings demonstrate that lentinan exerts its hepatoprotective effects primarily by suppressing key pathways involved in lipid synthesis and metabolism, thereby providing a molecular basis for the observed reduction in hepatic steatosis and improved liver function.

### 3.5 Integrative analysis reveals gut-liver crosstalk mediated by lentinan

In order to explore the potential molecular links underlying gut-liver crosstalk mediated by lentinan, we performed comprehensive correlation analyses between differentially abundant gut microbes, microbial metabolic pathways, and hepatic differentially expressed genes.

**Figure 5:**
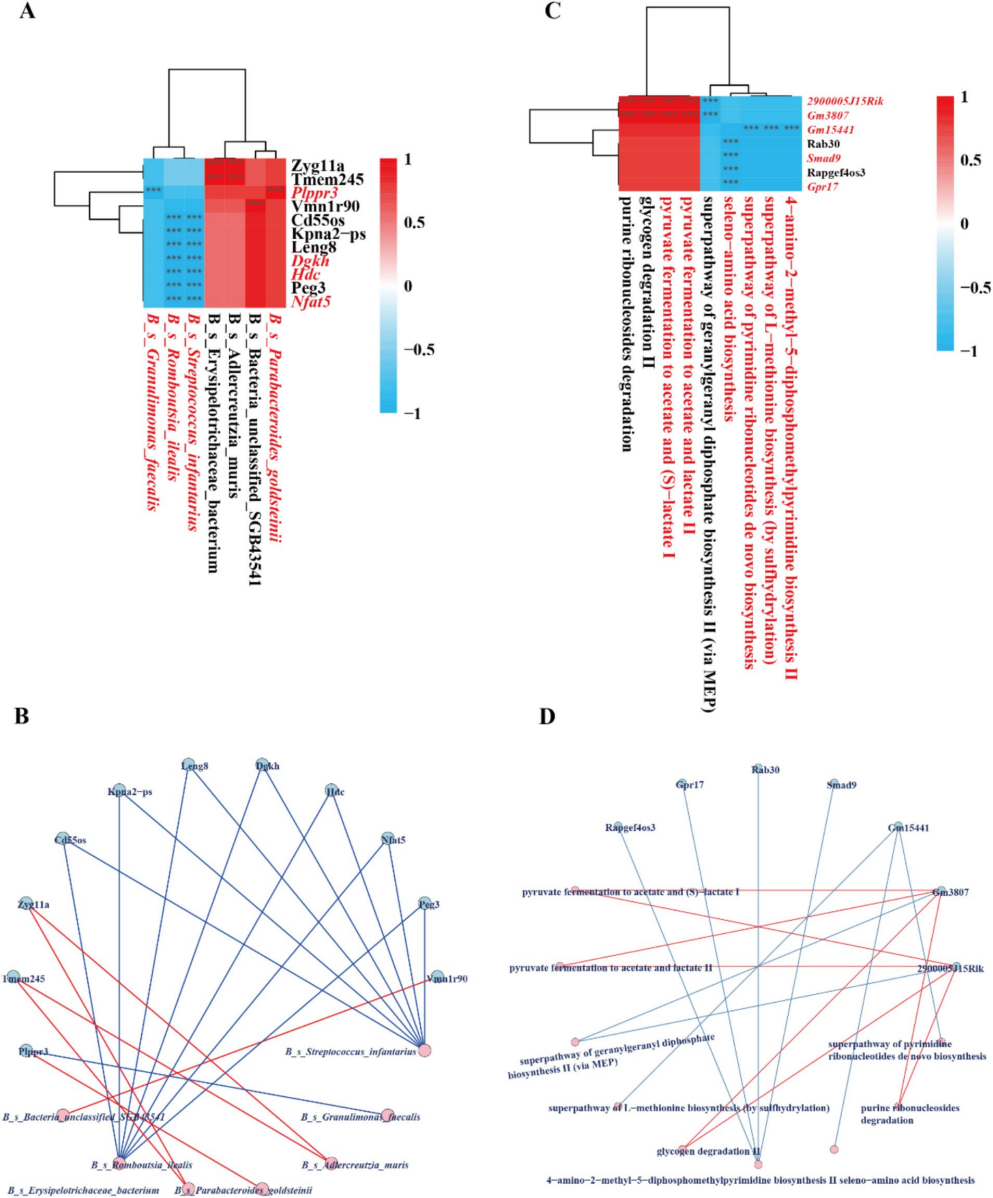
Correlation analysis between gut microbiota and hepatic differentially expressed genes. **(A)** Heatmap showing the correlation between differentially abundant bacterial genera and liver DEGs. Red indicates positive correlation, blue indicates negative correlation; asterisks denote statistical significance (*p ≤ 0.05, **p ≤ 0.01, ***p ≤ 0.001). **(B)** Network diagram illustrating significant correlations between specific gut bacteria and hepatic DEGs. Nodes represent bacterial species (red) or genes (blue), and edges represent significant correlations (positive: red lines; negative: blue lines). **(C)** Heatmap depicting correlations between enriched microbial metabolic pathways and liver DEGs. Pathways are listed on the x-axis, and DEGs on the y-axis. Color intensity reflects correlation strength. **(D)** Network diagram of significant associations between gut microbiota-derived metabolic pathways and liver gene expression. Nodes represent metabolic pathways (red) or DEGs (blue), with edge color indicating correlation direction (red: positive; blue: negative).

The heatmap analysis revealed specific and statistically significant correlations between bacterial taxa and hepatic gene expression (Figure 5A). Of particular interest, *P. goldsteinii*—a beneficial bacterium enriched by lentinan treatment—showed a strong positive correlation with *Plppr3* (*p* ≤ 0.001), a gene involved in lipid phosphate phosphatase activity and membrane lipid homeostasis. Conversely, *R. ilealis* and *S. infantarius*—both reduced by lentinan—demonstrated significant negative correlations with *Dgkh* (diacylglycerol kinase eta), a key enzyme in glycerolipid biosynthesis, and *Nfat5* (nuclear factor of activated T-cells 5), a critical regulator of *NF-κB* signaling and inflammatory responses (p ≤ 0.001 for all correlations). These findings suggest that lentinan-induced modulation of specific gut bacteria may directly influence hepatic lipid metabolism and inflammatory pathways. The correlation network further visualized these complex interactions (Figure 5B), highlighting *P. goldsteinii*, *R. ilealis* and *S. infantarius* as central hubs connecting multiple hepatic metabolic and inflammatory genes. This network organization supports the hypothesis that these bacterial taxa serve as key mediators of lentinan’s hepatoprotective effects.

Analysis of microbial metabolic pathways revealed additional layers of gut-liver communication (Figure 5C). Several immune-related genes (*Gpr17*, *Smad9*, *Gm15441*) (22, 23) exhibited significant negative correlations with microbial seleno-amino acid biosynthesis, L-methionine biosynthesis, and vitamin B1 precursor biosynthesis pathways (*p* ≤ 0.001), suggesting that lentinan-mediated reduction of these metabolic activities may contribute to decreased hepatic inflammation. Most intriguingly, pyruvate fermentation to acetate and lactate and glycogen degradation pathways—both enhanced by lentinan—showed strong positive correlations with two uncharacterized non-coding RNAs (*2900005J15Rik, Gm3807*) (*p* ≤ 0.001). These ncRNAs represent novel candidates for mediating microbial metabolite effects on host physiology. The pathway-gene interaction network (Figure 5D) confirmed these relationships, demonstrating dense connectivity between microbial amino acid metabolism pathways and host inflammatory genes, as well as between short-chain fatty acid production pathways and regulatory ncRNAs. This systems-level view strongly supports a model wherein lentinan reshapes gut microbiota composition and function, leading to altered production of bioactive metabolites that modulate hepatic gene expression networks involved in lipid metabolism, inflammation, and cellular homeostasis.

Collectively, these correlation analyses provide compelling evidence for specific microbe-host molecular interactions that may underlie lentinan’s therapeutic benefits against HFD-induced metabolic dysfunction.

## 4 Discussion

Our study demonstrates that lentinan, a bioactive polysaccharide derived from *Lentinus edodes*, exerts comprehensive protective effects against HFD-induced metabolic disorders through coordinated modulation of the gut-liver axis. Multi-dimensional analyses revealed that lentinan intervention effectively ameliorates dyslipidemia, hepatic steatosis, systemic inflammation, and intestinal barrier dysfunction in HFD-fed mice. Mechanistically, we established that lentinan’s therapeutic benefits are predominantly mediated through its ability to reshape gut microbiota composition and function, which subsequently reprograms hepatic gene expression networks governing lipid metabolism and inflammatory responses. This integrated mechanism distinguishes lentinan from conventional single-target therapies and positions it as a promising candidate for metabolic syndrome management.

Lentinan treatment induced remarkable taxonomic specificity in gut microbiota remodeling, with *P. goldsteinii* emerging as a key beneficiary showing substantial enrichment compared to HFD controls. This finding carries significant metabolic implications, as *P. goldsteinii* belongs to a genus recently recognized for its beneficial metabolic properties (24–26). Recent work by Wu et al. (27) demonstrated that polysaccharides from *H. sinensis* exert anti-obesity effects specifically through *P. goldsteinii* enrichment, enhancing adipose tissue thermogenesis and intestinal barrier integrity while reducing systemic inflammation—mechanisms that strikingly parallel the effects observed with lentinan in our study. Critically, we identified a significant positive correlation between *P. goldsteinii* abundance and hepatic *Plppr3* expression (Figure 5A), suggesting a direct molecular link between this bacterium and host lipid metabolism regulation. *Plppr3* (phospholipid phosphatase-related protein type 3) plays crucial roles in lipid phosphate phosphatase activity and membrane lipid homeostasis (28), with its upregulation potentially contributing to reduced hepatic lipid accumulation.

Conversely, lentinan treatment led to significant reductions in *A. muciniphila* and *R. ilealis*, taxa whose roles in metabolic health are highly context-dependent. Notably, in our model, *A. muciniphila* abundance showed no significant difference between HFD and LFD groups, and lentinan-induced reduction of this taxon occurred concurrently with comprehensive metabolic improvements—including attenuated weight gain, reduced hepatic steatosis, and restored intestinal barrier integrity—challenging the notion of its uniformly beneficial role. This aligns with accumulating evidence that *A. muciniphila*’s effects are context-dependent rather than universally beneficial. A randomized trial (NCT04797442) demonstrated that supplementation showed significant reductions in body weight, fat mass, and HbA1c only in individuals with low baseline abundance, with no efficacy in those harboring high baseline levels—a pattern corroborated in germ-free mouse colonization studies (29). Further underscoring its functional duality, pasteurized *A. muciniphila* was reported to upregulate GP2 in intestinal microfold cells, potentially increasing susceptibility to *Salmonella* infection (30). Collectively, these findings indicate that *A. muciniphila*’s impact is contingent on host microbial context and physiological state. In our HFD-induced metabolic stress model, lentinan-mediated reduction of *A. muciniphila* likely reflects a targeted recalibration of the gut ecosystem toward a configuration favoring metabolic homeostasis, rather than depletion of a presumed “beneficial” taxon. *R. ilealis*, a member of the Clostridiaceae family associated with inflammatory conditions and impaired barrier function (31), exhibited significant negative correlations with both *Dgkh* (involved in glycerolipid biosynthesis) and *Nfat5* (a regulator of osmotic stress and inflammation) (Figure 5A), providing a potential mechanistic explanation for lentinan’s lipid-lowering and anti-inflammatory effects (32, 33). These findings collectively suggest that lentinan’s efficacy operates through a sophisticated rebalancing of the gut ecosystem rather than simple enrichment of conventionally beneficial taxa.

The lentinan-induced microbial shifts correlated strongly with enhanced intestinal barrier integrity, evidenced by significantly increased claudin-1 expression in colonic epithelium (Figure 2L). Claudin-1 is a key tight junction protein essential for maintaining intestinal permeability barrier and preventing bacterial translocation. While *A. muciniphila* is often associated with barrier enhancement through mucin degradation and renewal (34), our data suggest that in HFD-induced metabolic stress, the combined effects of *Parabacteroides* enrichment and reduction of potentially inflammatory taxa like *R. ilealis* provide superior barrier protection. This hypothesis aligns with emerging evidence that specific combinations of bacterial taxa, rather than individual species, determine overall barrier function through synergistic effects on tight junction protein expression (35). The concurrent enrichment of *P. goldsteinii* and increased claudin-1 expression likely compensates for *A. muciniphila* reduction, highlighting that community functionality emerges from network interactions rather than individual species contributions—a perspective that supports the development of precision microbiome therapeutics accounting for baseline microbial configurations.

Hepatic transcriptomic analysis revealed significant downregulation of glycerolipid metabolism and oxidative phosphorylation pathways following lentinan intervention (Figures 4A–C; *p* ≤ 0.01). These transcriptional changes spatially and temporally correlated with reduced hepatic lipid accumulation observed histologically (Figures 1L,M), and integrated analysis with metagenome data showed significant correlations between specific bacterial taxa and hepatic gene expression patterns (Figure 5), suggesting coordinated gut-liver regulation. Multi-dimensional assessment confirmed lentinan’s hepatoprotective effects, with significant reductions in serum ALT and AST levels alongside normalized CHE activity and LDH levels (Figures 2H–K). These biochemical improvements directly paralleled histological evidence of reduced steatosis and improved hepatic architecture (Figures 1L,M). The concordance between transcriptional reprogramming, functional biomarkers, and morphological improvements demonstrates lentinan’s dual action—direct hepatic regulation combined with microbiome-mediated effects—providing a multi-target therapeutic approach distinct from conventional single-pathway pharmacotherapies.

**Figure 6:**
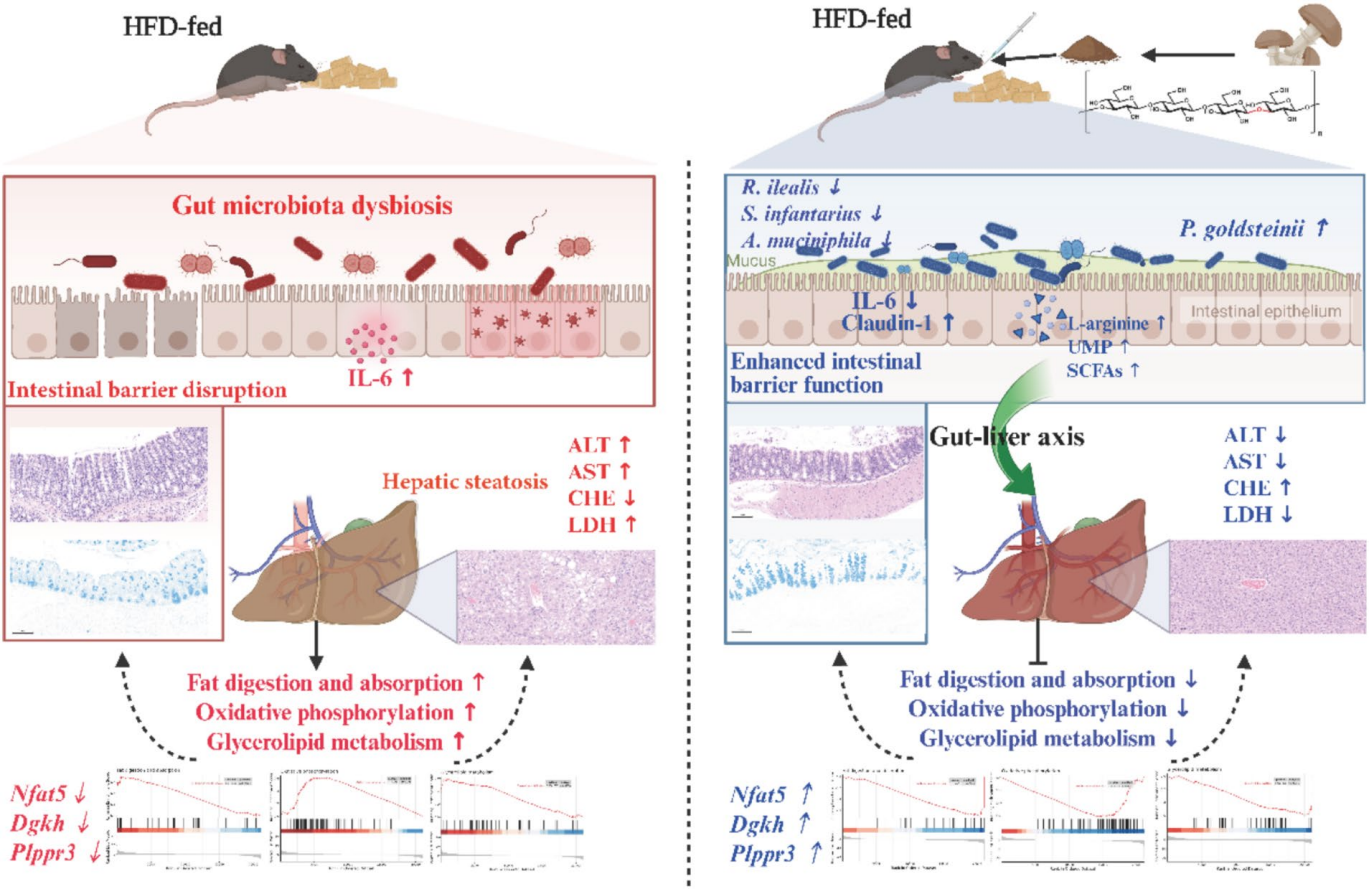
Schematic representation of the mechanism by which lentinan ameliorates high-fat diet-induced metabolic dysfunction through precision modulation of the gut-liver axis. Lentinan administration effectively alleviates diet-induced metabolic abnormalities, including excessive weight gain, systemic inflammation, intestinal barrier disruption, and hepatic steatosis. These therapeutic effects are mediated through selective enrichment of *P. goldsteinii*, enhancement of microbial L-arginine and UMP biosynthesis pathways, restoration of intestinal epithelial integrity via claudin-1 upregulation, and coordinated reprogramming of hepatic lipid metabolism. The gut microbiota-derived metabolites communicate with the liver through the portal circulation, establishing a functional gut-liver axis that ultimately resolves metabolic dysfunction.

This study advances the field by demonstrating that lentinan functions not merely as a conventional prebiotic that restores a generic “lean microbiota” profile, but as a precision modulator that reprograms specific microbial metabolic functions—particularly amino acid biosynthesis pathways—that directly interface with host inflammatory and lipid metabolism regulation (36) (Figure 6). Notably, our metagenomic analysis revealed significant enrichment of L-arginine biosynthesis pathways I/II and UMP biosynthesis pathways I/II/III in the gut microbiome following lentinan intervention (Figure 3J). These findings gain critical physiological relevance from clinical and experimental evidence: population studies demonstrate L-arginine supplementation significantly improves metabolic parameters in obese individuals (37), while animal models reveal its capacity to promote brown adipose tissue conversion (38, 39), enhance mitochondrial activity (40), and activate thermogenic metabolism (41). Similarly, recent research established an inverse correlation between UMP concentration in human milk and childhood obesity, with experimental validation showing UMP suppresses fat accumulation through promoting mitochondrial biogenesis and thermogenic metabolism (42). Together, these lentinan-induced microbial metabolic shifts provide a mechanistic foundation for the observed improvements in host energy homeostasis. Unlike previous lentinan research focused on antitumor and immunomodulatory effects (43, 44), our integrated multi-omics approach reveals its unique application in metabolic disorders through targeted remodeling of the gut-liver axis. This work establishes a conceptual framework where functional microbial pathway modulation, rather than simple taxonomic composition changes, underlies therapeutic efficacy—a paradigm that reconciles seemingly contradictory findings in microbiota-targeted therapies. Clinically, lentinan represents a promising candidate for metabolic disorder management due to its dual regulation of gut microbiota and hepatic lipid metabolism, with potential for personalized application based on baseline microbial configurations and synergistic use with conventional therapies like metformin.

This study has several limitations that warrant consideration. While our integrated analyses provide compelling associative evidence for gut-liver interactions, future studies incorporating microbial metabolite profiling, germ-free mouse models, or fecal microbiota transplantation experiments will be necessary to establish causality. Additionally, our investigation was limited to a single lentinan dose and timepoint, necessitating comprehensive dose–response and time-course studies to optimize therapeutic protocols. Future work should focus on lentinan’s active components, mono-colonization experiments with key strains (e.g., *P. goldsteinii*), and targeted metabolite supplementation studies (particularly acetate/lactate) to establish mechanistic causality. Third, this study is the lack of protein-level validation of the identified transcripts, which will be addressed in future investigations to confirm the translational relevance of our transcriptomic findings. Ultimately, our findings establish lentinan as a potent modulator of the gut microbiota-liver axis that ameliorates metabolic dysfunction through coordinated microbial and transcriptional reprogramming, highlighting the therapeutic potential of targeting host-microbe metabolic crosstalk for treating obesity-related disorders, though rigorous human clinical trials are necessary before translational application.

## 5 Conclusion

In conclusion, this study establishes lentinan as a precision modulator of the gut-liver axis that effectively ameliorates diet-induced metabolic dysfunction through taxonomically selective remodeling of gut microbiota—particularly enrichment of *P. goldsteinii*—and subsequent reprogramming of hepatic lipid metabolism pathways. Unlike conventional prebiotics that aim to restore a generic “lean microbiota” profile, lentinan functions by specifically reprogramming microbial metabolic functions, particularly L-arginine and UMP biosynthesis pathways, which interface with host inflammatory regulation. This mechanism, revealed through integrated multi-omics analyses, provides a conceptual framework that reconciles seemingly contradictory findings in microbiota-targeted therapies and offers a methodological blueprint for dissecting mechanism-specific host–microbe interactions. While causal validation in germ-free models and clinical translation require further investigation, our findings highlight lentinan’s therapeutic potential as a multi-target agent that simultaneously enhances intestinal barrier integrity, reduces systemic inflammation, and improves hepatic steatosis, positioning it as a promising candidate for precision microbiome-based interventions in metabolic syndrome management.

## Data Availability

The raw sequence data generated during this study have been deposited in the CNCB Genome Sequence Archive (GSA) under the accession CRA036737 and CRA036704 (https://ngdc.cncb.ac.cn/gsa). All other relevant data supporting the findings of this study are available from the corresponding author upon reasonable request.
