# Supplementary material for: Lentinan alleviates metabolic dysfunction implicating Parabacteroides goldsteinii-enriched gut microbiota and hepatic lipid metabolism reprogramming through gut-liver axis-associated mechanisms
Source: Front Nutr. 2026 Jul 1;13:1841358. doi: 10.3389/fnut.2026.1841358 (PMC13371872; doi:10.3389/fnut.2026.1841358)
Supplement: Supplementary file 1 [file Data_Sheet_1.DOCX]

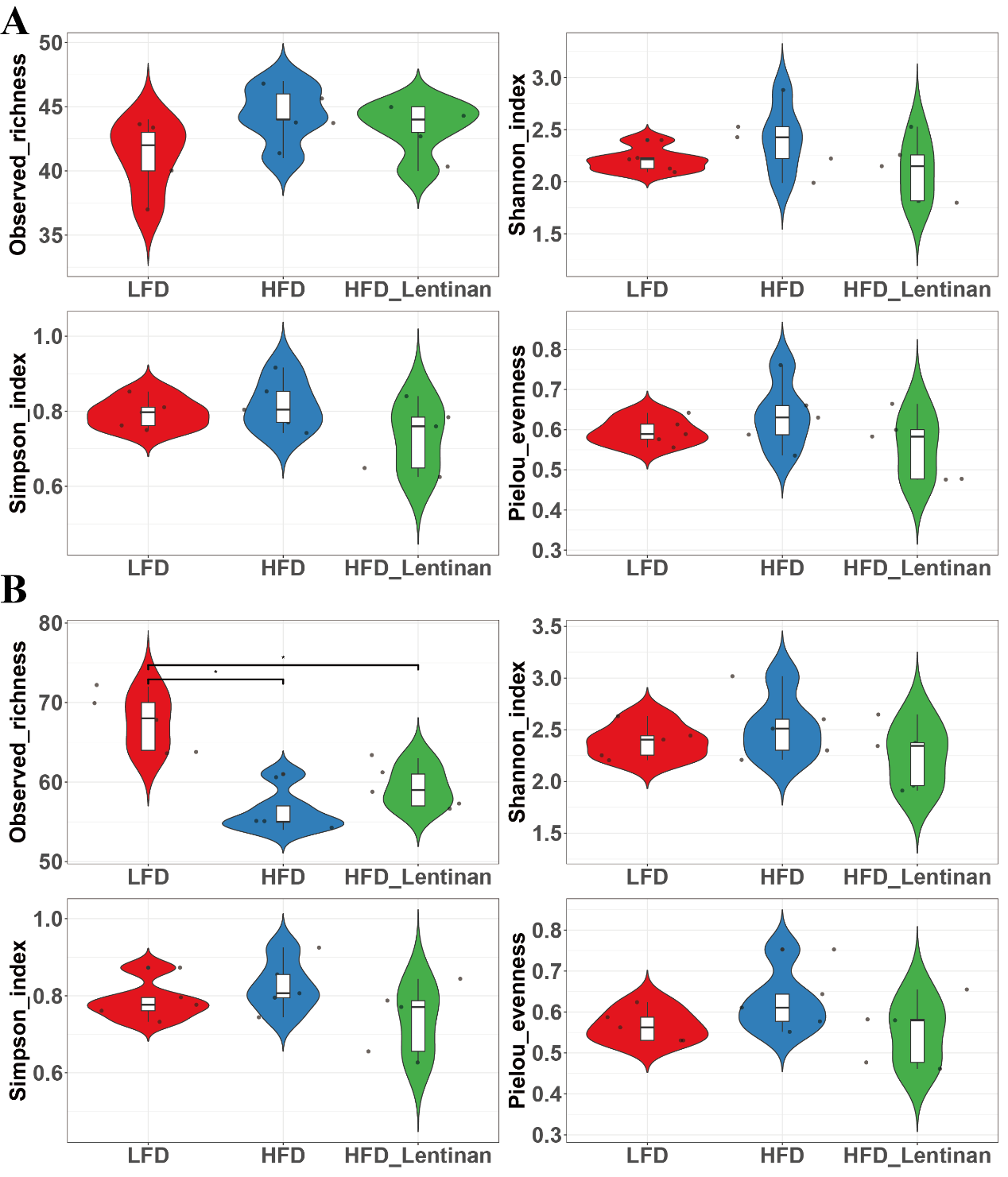


**Figure S1.** **Alpha diversity analysis of the gut microbiota.** Observed richness, Shannon index, Simpson index and Pielou evenness at the genus (A) and species (B) level.


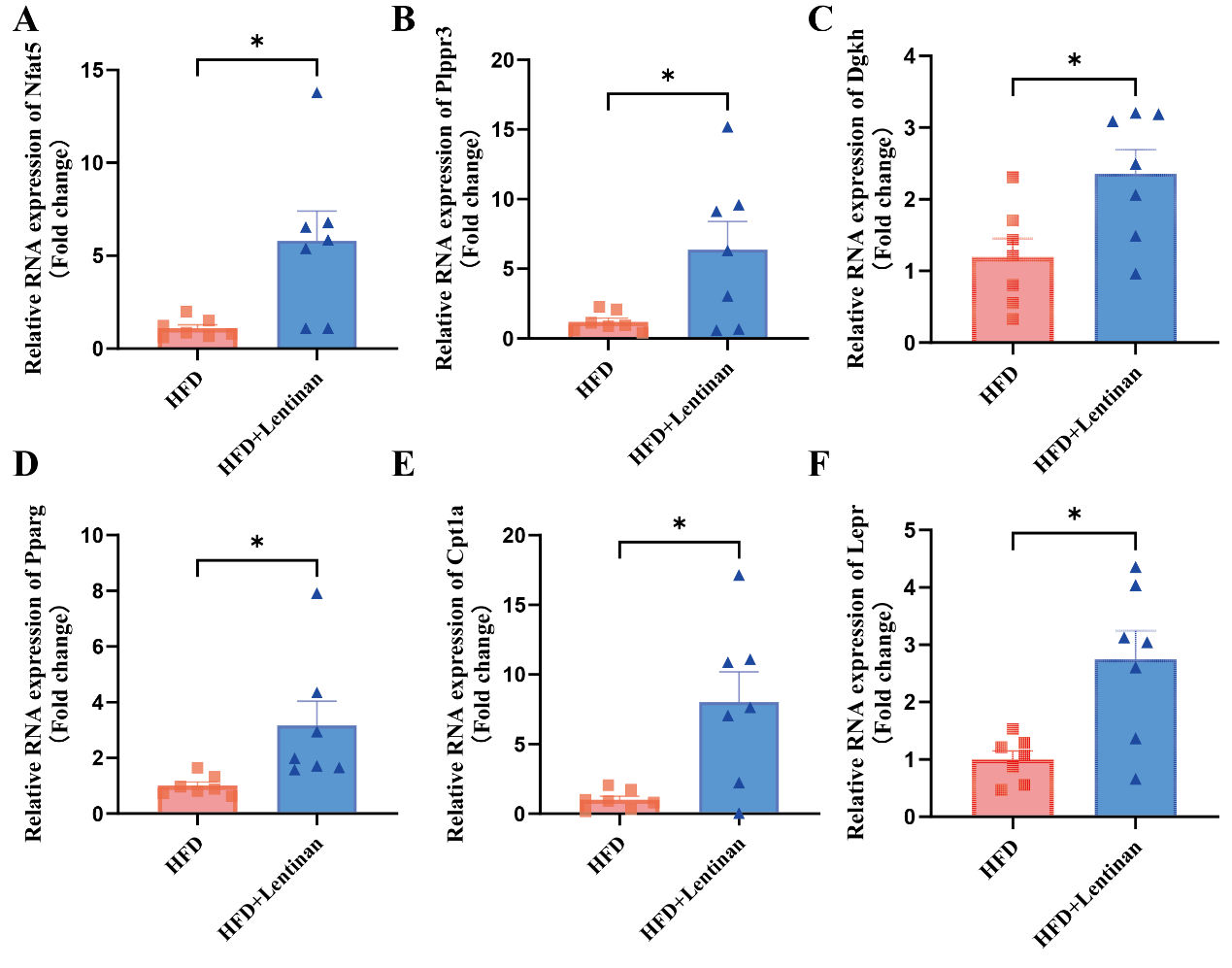


**Figure S2. qPCR validation of key differentially expressed genes associated with liver metabolic regulation.** mRNA expression of *Nfat5* (A)*, Plppr3* (B)*, Dgkh* (C)*, Pparg* (D)*, Cpt1a* (E), *Lepr* (F).
